# The Sharp Rise in the Use of Low- and No-Calorie Sweeteners in Non-Alcoholic Beverages in Slovenia: An Update Based on 2020 Data

**DOI:** 10.3389/fnut.2021.778178

**Published:** 2021-11-19

**Authors:** Edvina Hafner, Igor Pravst

**Affiliations:** ^1^Nutrition Institute, Nutrition and Public Health Research Group, Ljubljana, Slovenia; ^2^Biotechnical Faculty, University of Ljubljana, Ljubljana, Slovenia; ^3^VIST–Higher School of Applied Sciences, Ljubljana, Slovenia

**Keywords:** low-and no-calorie sweeteners, beverages, reformulation, added sugar, food composition, database

## Abstract

Reducing added sugars in non-alcoholic beverages is an important public health goal, which can result in increased use of low- and no-calorie sweeteners (LNCS). The aim of this study was to investigate recent changes in the use of LNCS in non-alcoholic beverages in the Slovenian food supply. The national branded foods dataset was updated with beverages available in 2020, and compared with previous datasets. The data were extracted from food labels. In 2020, *N* = 1,650 unique beverages were found in shops from five different retailers, covering the majority of the national market. The use of LNCS increased from 13.2% in 2017 and 15.5% in 2019 to 20.2% in 2020, with a major growth in soft drinks (16.8, 19.6, and 26.7%, respectively). We observed a significant growth of beverages containing both LNCS and added sugar. Results were also consistent with sales data, which showed that increased offer of beverages with LNCS also resulted in similarly increased sales of such beverages. The average energy and total sugar content in non-alcoholic beverages decreased, which reflects both the higher percentage of beverages with LNCS, and also the reduction of the sugar content in beverages with only added sugar. Analyses of product-specific reformulation practices highlighted reduced sugar content in 16.8% of products, and in 3.6% with the use of LNCS. The most commonly used LNCS are acesulfame K, sucralose, and aspartame. Typically, combinations are used, however steviol glycosides, sucralose and saccharin are also used alone, in most cases combined with added sugar. The results indicated rapid changes in the use of LNCS in non-alcoholic beverages in the Slovenian food supply, making further monitoring of this area highly relevant.

## Introduction

Low- and no-calorie sweeteners (LNCS) are a group of food additives that provide a sweet taste with no or fewer calories per gram of food, compared to sugar (1). They include both intensive sweeteners and polyols, which can partially or entirely replace sugar in various foods and drinks (2). Since excessive sugar intake is a major public health issue in the modern diet, its reduction is a key step in fighting against the worldwide obesity epidemic (3). The World Health Organization (WHO) recommends keeping free sugar consumption below 10% of the daily energy intake, and preferably even below 5% (4, 5).

Sugar-sweetened beverages are one of the major contributors to sugar intake (6–8), with sugar often being the only source of energy (9). Reduction of the intake of sugar-sweetened beverages is considered an important public health goal (10). In addition to raising public awareness, national authorities also encourage food manufacturers to reformulate their products to reduce added sugars. Some governments have implemented a sugar/soda tax (e.g., UK, Ireland, France, Mexico), and already results show a rapid increase in reformulation and decreased sales of sugary drinks (11–13). Since sugar is an important ingredient that provides a desired taste, its reduction could decrease consumers' interest in the product. Therefore, LNCS are often a convenient alternative, allowing formulation of products with reduced sugar and energy content, while achieving the same level of sweetness (3, 14). Consumer awareness and reformulation activities can result not only in increased availability, but also in increased intake of products with added LNCS (2, 15, 16). Although the use of LNCS is carefully regulated (17, 18), some studies have suggested possible risks related to the excessive consumption of LNCS, i.e., impacts on the microbiome (19), and increased risk of metabolic syndrome (20) and diabetes (21). However, it should be noted that because different LNCS have very different chemical structures, generalizations of health risks are not appropriate (22, 23).

With consideration of abovementioned issues, careful monitoring of changes in the food supply is crucial. Out of all foods and drinks, non-alcoholic beverages have been shown to be one of the major contributors to LNCS availability (15) and intake (24, 25). In Slovenia, monitoring of the use of LNCS in non-alcoholic beverages was undertaken for products available in 2017, following by further data collection in 2019 (26). While no significant differences were observed in a comparative study (26), signs of increased use of LNCS were found, making this area very interesting for further research. In Slovenia, there are currently no legislative restrictions or taxations for the content of sugars in sugary drinks. However, the government is promoting a reduction of added sugar through mass media and voluntary pledges (27). Although food reformulation in general is encouraged, the National Program on Nutrition and Health Enhancing Physical Activity 2015–2025 (28) specifically mentions that the use of LNCS should be also reduced. Given that a major part of the Slovenian food market consists of branded foods imported from other countries, the monitoring of the food supply will provide important insights into the European food supply. Furthermore, such insights are very important nationally for efficient, evidence-based policy decisions in the future.

Monitoring of the composition of the foods in the food supply is very challenging, because thousands of different products are available on the market, their compositions can change, and notable differences can be observed in different regions (29). This complicates data collection, and available datasets are commonly focused on nutritional food components, which are part of mandatory nutrition declarations, and less commonly on other food constituents, such as additives. It should also be mentioned that nationally representative branded food datasets are only available in some countries, and that different data collection approaches are used. A standard approach is cross-sectional food monitoring studies in food stores, where data is extracted from food labels. This methodological approach was harmonized within the Global Food Monitoring Group (30) and INFORMAS initiative (31, 32). In Slovenia, food monitoring studies are conducted within the government funded national research programme “Nutrition and Public Health,” and are supported by the HORIZON2020 “Food Nutrition Security Cloud” project (FNS-Cloud; www.fns-cloud.eu), which is funded by the European Commission.

The present study aimed to investigate recent changes in the use of LNCS in non-alcoholic beverages in the Slovenian food supply. The national branded foods dataset was updated with beverages available in 2020. The dataset, containing both nutrition declaration and ingredients data, was compared with previous datasets compiled in 2017 and 2019. The use of repeated cross-sectional studies also enabled investigation of product-specific reformulation practices.

## Materials and Methods

### Data Collection and Processing

This repeated cross-sectional study used new data collected in Slovenia in 2020, and previously reported data collected in 2017 and 2019 (26). In all 3 years, the data were collected using the Composition and Labeling Information System (CLAS, Nutrition Institute, Ljubljana, Slovenia) (33). In Slovenia, CLAS collect information on prepacked foods and drinks available at major retailers that represent most of the market share. The details regarding data collection are described elsewhere (34). In brief, pictures of all pre-packed foods and drinks with a unique European Article Number (EAN) barcode available at the time of sampling were collected. All the information on the nutritional composition and ingredients needed for the study were extracted from photographs using the online CLAS tool. The 2020 data were collected from all major retailers with nationwide networks of shops. The following shops in Ljubljana (Slovenia) were included in the data collection: two mega markets (Mercator Center, Interspar), two supermarkets (Tuš, Spar), and three discount markets (Hofer, Lidl, Eurospin). With the exception of Eurospin, the same shops were also included in the data collection in 2017 and 2019. Products were classified based on previously developed global categorisations by Dunford et al. (35), with minor adaptations considering the specifics of the European market. This study examined five categories of non-alcoholic beverages: juices, nectars, soft drinks, energy drinks, and sports drinks. Details regarding product categorization have been previously described (26).

The LNCS and added sugar were identified from the ingredient list on the food packaging. We examined the use of all 19 LNCS that are currently authorized for use in food products in European regulations (35). LNCS were identified by their name and/or their E number. Similarly, we identified added sugar, which was defined as all mono- and disaccharides added to foods, excluding fruit juices and purees. Based on this, beverages were segmented into four groups: (1) with added LNCS; (2) added sugar; (3) with added LNCS and added sugar; (4) without added LNCS and without added sugar. To further examine these four groups with consideration of market-share differences, we compared the availability of such products in the food supply with sales data. We were able to obtain nation-wide 12-month sales data for 2017 and 2020 from retailers, representing over 50% of the national food supply. Sales data was provided in universal form including EAN barcode number, number of products sold per year, and package quantity (L). We matched products in the food supply dataset with those in sales data dataset. EAN barcode numbers were used as unique product identifiers for the matching process. There were 705 (68%) matches found in the 2017 dataset, and 1,007 (61%) in the 2020 dataset.

To provide insights into reformulation practices, we also matched products (using EAN barcode numbers) in the 2020 dataset with those in the 2017/2019 datasets. A similar approach was used by Bernstein et al. (36). All products available in the 2020 dataset were searched for matches in the 2017/2019 datasets. For those matches, we calculated the difference in total sugar content (TSC) between 2017 and 2020. If a product was not available in the 2017 dataset, we used the sugar content from the 2019 dataset.

### Statistical Analysis

Data were collected and processed using the Composition and Labeling Information System (CLAS) (Nutrition Institute, Ljubljana, Slovenia) and Microsoft^TM^ Excel 2019. Statistical analysis was performed using IBM SPSS v.26. Descriptive statistics were used for reporting the prevalence of beverages with added LNCS, added sugar, both, or neither. We also assessed the prevalence of individual LNCS and their combinations. A two-tailed z-test was used to compare changes in the LNCS use between time periods. A t-test was used to compare the mean energy value (EV) and TSC within each category between time periods. For sale-weighting, we calculated the total amount (L) of beverages sold per year (separately for years 2017, and 2020) using package quantity (L) and the number of sold products. We presented the sale-weighted proportions and compared them with the food supply offer (products available at the time of sampling).

## Results

### Presence of LNCS and Their Effect on Energy Value and Total Sugar Content

The 2020 dataset consisted of *N* = 1,650 non-alcoholic beverages. The between category distribution was similar to that observed in 2017 and 2019 (26). Soft drinks had the largest share in the sample (*N* = 898; 54.5%), followed by juices (*N* = 350; 21.2%), nectars (*N* = 229; 13.8%), energy drinks (*N* = 136; 8.2%), and sports drinks (*N* = 37; 2.2%) (Table 1). At least one LNCS was present in 333 products. The proportion of products with LNCS therefore increased from 15.5% in 2019 to 20.2% in 2020 (*p* < 0.01). The biggest difference between 2019 and 2020 was observed in soft drinks; the LNCS beverages increased from 19.6% to 26.7% (*p* < 0.01). A comparison with the 2017 data also provided interesting insights. In energy drinks, the proportion of products with LNCS increased between 2017 and 2019 (from 16.9% to 41.8%) (26), and stayed at a comparable level in 2020 (41.2%). Interestingly, the overall number of nectars increased considerably (from 135 in 2017 to 229 in 2020), but the number of such products with LNCS stayed almost the same (*n* < 20), which resulted in a decreased proportion of nectars with LNCS (*p* < 0.05). Beverages with LNCS are most often sports (67.6%) and energy drinks (41.2%), but due to the small number of products, they do not contribute much to the overall supply of non-alcoholic beverages with LNCS. No use of LNCS was observed in juices.

**Table 1:** Sample description and availability of non-alcoholic beverages with added low- and/or no-calorie sweeteners (LNCS) in the Slovenian food supply in 2017, 2019, and 2020.

	**2017**		**2019**		**2020**	
	**Total**	**Added LNCS**	**Total**	**Added LNCS**	**Total**	**Added LNCS**
	***N* (%)**	***N* (%)**	***N* (%)**	***N* (%)**	***N* (%)**	***N* (%)**
Total	1,043 (100)	138 (13.2)	1,221 (100)	189 (15.5)	1,650 (100)	333 (20.2)^a, b^
Soft drinks	555 (53.2)	93 (16.8)	601 (49.2)	118 (19.6)	898 (54.5)	240 (26.7)^a, b^
Juices	267 (25.6)	/	330 (27.0)	/	350 (21.2)	/
Nectars	135 (12.9)	16 (11.9)	158 (12.9)	11 (7.0)	229 (13.8)	12 (5.3)^b^
Energy drinks	65 (6.2)	11 (16.9)	110 (9.0)	46 (41.8)	136 (8.2)	56 (41.2)^b^
Sports drinks	21 (2.0)	18 (85.7)	22 (1.8)	14 (63.6)	37 (2.2)	25 (67.6)

*N—number of products; Data for 2017 and 2019 from Hafner et al. (26).*

*^a^significant change between 2019 and 2020.*

*^b^significant change between 2017 and 2020*.

The results of the segmentation of beverages based on added sugar and/or LNCS are presented in Table 2. The largest contribution to the increase in the use of LNCS was made by beverages that use a combination of LNCS and added sugar. In 2017 and 2019, the proportions of such products were 7.6 and 7.5, respectively, increasing to 11.0% in 2020 (*p* < 0.01, comparison with 2017 data). Notable changes were also observed in the use of LNCS alone (5.7, 7.9, and 9.2, respectively).Similar trends were also observed, when 12-months sales values were considered, to account for market-share differences between different beverages in the food supply (Supplementary Figure 1). Increased availability of beverages with LNCS was also reflected in the increased sales of such beverages. From 2017 to 2020 the sale-weighted proportion of beverages with LNCS increased from 10.8 to 18.2%. Sales increased both for beverages with added only LNCS (from 4.1 to 8.8%) as well as for beverages with added LNCS and sugar (from 6.7 to 9.4%). The largest market share represented beverages with added sugar, but their share fell from 81.5% in 2017 to 72.2% in year 2020, when notably increased sales of beverages with LNCS were observed. Beverages without added sugar and LNCS, despite representing a large proportion of the available beverages, represented only 7.7% of the volume sales-market in 2017, and 9.6% in 2020.

**Table 2:** Comparison of the energy value and total sugar content in non-alcoholic beverages, based on the presence of added sugar and low and/or no-calorie sweeteners (LNCS).

	**Added sugar**	**Added LNCS**	**2017**	**2019**	**2020**	**Energy value (kJ/100 mL)**			**Total sugar content (g/100 mL)**		
						**2017**	**2019**	**2020**	**2017**	**2019**	**2020**
			***N* (%)**	***N* (%)**	***N* (%)**	**Mean (SD)**	**Mean (SD)**	**Mean (SD)**	**Mean (SD)**	**Mean (SD)**	**Mean (SD)**
Total			1043 (100%)	1221 (100%)	1650 (100%)	152.2 (66.5)	147.9 (70.9)	140.1 (72.2)***	8 (3.6)	7.6 (3.8)	7.3 (3.9)***
	**–**	**–**	300 (28.7%)	398 (32.6%)	435 (26.4%)	174.8 (69.1)	174.9 (67.6)	179.9 (91.7)	8.3 (3.8)	8.2 (3.8)	8.3 (3.9)
	✓	**–**	605 (58.0%)	634 (52.9%)	882 (53.5%)	161.8 (50.8)	159 (52.7)	154 (54)**	8.9 (2.7)	8.7 (2.8)	8.5 (3)**
	**–**	✓	59 (5.7%)	97 (7.9%)	152 (9.2%)	22.3 (37.9)	14.3 (27.9)	13 (20.3)	1 (2.1)	0.4 (1.4)	0.3 (1)*
	✓	✓	79 (7.6%)	92 (7.5%)	181 (11.0%)**	92.8 (28.1)	96.2 (40)	98.4 (41.1)	4.8 (1.5)	5.1 (2.3)	5.3 (2.3)
Soft drinks			555	601	898	126.8 (61.8)	119.8 (63.3)	109*** (61.2)	7 (3.5)	6.5 (3.5)	6 (3.5)***
	**–**	**–**	26	43	45	60.2 (66.8)	70 (55.4)	40.8 (51.5)	2.5 (3.5)	2.4 (2.5)	1.5 (2.3)
	✓	**–**	436	440	613	146.3 (48.4)	142.5 (50.9)	134.8** (47.4)	8.2 (2.7)	7.9 (2.7)	7.6 (2.7)**
	**–**	✓	39	50	105	4.4 (4.8)	5.8 (8)	9.4** (14.6)	0.1 (0.2)	0.2 (0.8)	0.2 (0.6)**
	✓	✓	54	68	135	90.4 (29.7)	87.5 (35.1)	91.9 (33.8)	4.9 (1.6)	4.7 (2)	5 (1.9)
Juices	**–**	**–**	267	330	350	186.7 (57)	190.5 (54.8)	193 (52.8)	8.8 (3.3)	9.1 (3.1)	9.4 (2.9)*
Nectars			135	158	229	190 (45.7)	184.8 (46.9)	190.4 (50.2)	10 (2.4)	9.5 (2.8)	9.6 (3.3)
	**–**	**–**	5	23	39	135 (107.5)	141.8 (75)	145.4 (68.7)	7.8 (6.6)	6.8 (4.3)	5.9 (4.1)
	✓	**–**	114	124	178	203.3 (29.1)	198.8 (28.6)	204.6 (33.7)	10.6 (1.6)	10.4 (1.8)	10.8 (2.1)
	**–**	✓	10	6	5	104.7 (5.4)	116.5 (23.1)	91.6 (29.5)	5.6 (0.4)	5.2 (0.7)	4.6 (1.6)
	✓	✓	6	5	7	125.5 (29.1)	117.2 (16)	105.7 (14.7)	6.5 (1.6)	6 (0.6)	5.7 (0.4)
Energy drinks			65	110	136	174.2 (72.9)	135.6 (92.9)	141.5 (88.9)**	9.6 (4.2)	7.4 (5.5)	7.7 (5.2)**
	**–**	**–**	2	2	1	240	203 (52.3)	224	13	11.4 (2.3)	12.5
	✓	**–**	52	62	79	203.8 (28.7)	203.1 (31.4)	197.5 (40.6)	11.4 (1.6)	11.3 (1.8)	11 (2.3)
	**–**	✓	8	36	35	11.5 (4.7)	10.6 (7.2)	12.2 (6.1)	0 (0)	0 (0)	0 (0)
	✓	✓	3	10	21	72.7 (7.2)	153.9 (45.6)	142.4 (70.1)	3.7 (0.4)	8.3 (2.7)	7.8 (3.9)
Sports drinks			21	22	37	85.1 (30.9)	74.2 (41.5)	81.3 (38.3)	3.9 (1.4)	3.5 (2.1)	3.7 (2)
	✓	**–**	3	8	12	102.7 (26.5)	104.5 (16.3)	103.1 (24.1)	5.1 (1.2)	5.3 (0.7)	5.2 (0.9)
	**–**	✓	2	5	7	2.5 (3.5)	4.6 (2.6)	13.7 (21.5)	0 (0)	0 (0)	0 (0)
	✓	✓	16	9	18	92.1 (12.3)	86 (13.7)	93.1 (14.4)	4.2 (0.4)	3.9 (0.2)	4.2 (0.4)

*N, number of products; SD, standard deviation.*

*p < 0.05; **p < 0.01; ***p < 0.001. Data for 2017 and 2019 from Hafner et. al (26).

We also observed that the energy and sugar content of the beverages also changed in the last few years (Table 2). Taking the whole 2020 dataset into account, the average content of energy and sugar in the non-alcoholic beverages was 140.1 kJ and 7.3 g/100 mL, respectively. The EV and TSC therefore significantly fell from 2017 (152.2 kJ and 8 g of sugar per 100 mL; *p* < 0.001) (Table 2). This reduction is the result of both increased use of LNCS and a reduction in TSC in other beverages. The results showed that both the EV and the TSC were considerably reduced when the sugars were partially replaced by LNCS, while the difference was even more pronounced when only LNCS were used for sweetening. Encouragingly, a decrease in the EV and TSC was also observed in beverages with only added sugar (*p* < 0.01). The amount of TSC also dropped slightly in beverages with only added LNCS (*p* < 0.05). In individual categories, considerable changes were observed in soft drinks, which, due to their abundance, contributed the most to the changes in the overall sample. The mean EV and TSC in soft drinks decreased both in the whole category (*p* < 0.001), and also specifically in soft drinks with only added sugar (*p* < 0.01). In other categories, we observed a decrease in the mean EV and TSC for energy drinks (*p* < 0.01), which was mostly due to the increased use of LNCS, as after segmentation based on added LNCS and added sugar, no differences were observed in the four segments. A slight increase in TSC was also observed in juices (*p* < 0.05), which could mean that the supply of juices from sweeter fruits is on the rise. Juices were also the category with the highest EV and TSC.

**Figure 1:**
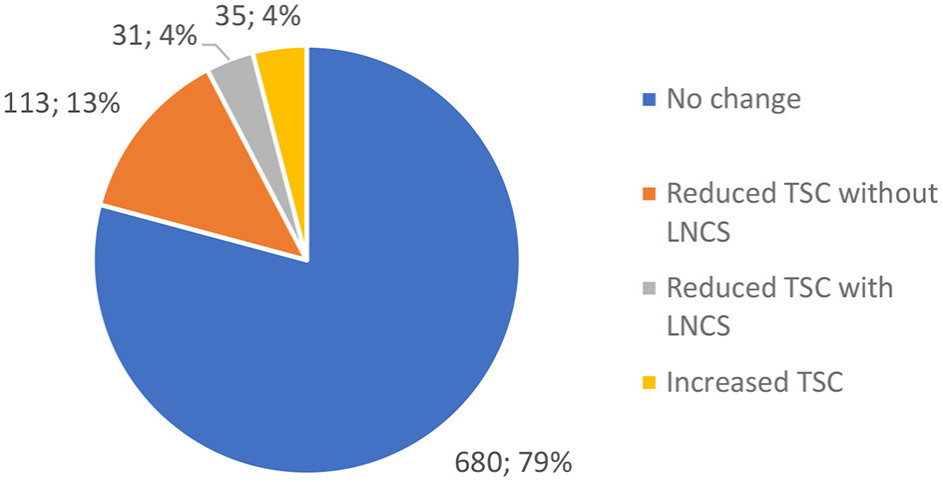
Changes in the total sugar content (TSC) in beverages available in 2020, which were also found in the 2017/2019 dataset (*N* = 859; LNCS–low- and/or no-calorie sweeteners).

The food matching method was used to provide insights into reformulation practices in specific products. Analyses was undertaken with *N* = 859 non-alcoholic beverages in the 2020 dataset, for which matches were found in previous datasets. For 680 (79.2%) products, no change in TSC was observed. Out of 179 products with changed compositions, 144 (16.8%) showed a reduction and 35 (4.1%) an increase in TSC (Figure 1). It should be noted that 31 (3.6%) products with reduced TSC also contained LNCS, and 20 (2.3%) did not contain LNCS in previous years. Most products with reduced sugar content were in the category of soft drinks (*N* = 107); this trend was particularly notable in fruit drinks. Interestingly, the average TSC in sugar-reduced reformulated beverages was quite high (7.4 g per 100 mL, in comparison to the overall 2020 average of 7.3 g per 100 mL). The mean sugar reduction for products without LNCS was −1.1 g per 100 ml, and −3.3 g for products with added LNCS. Products reformulated with increased TSC had on average 1.0 g more sugar per 100 ml. Interestingly, we found four energy drinks that did not contain LNCS in previous datasets, where LNCS were added in 2020, but their sugar content remained the same.

### Prevalence of Different LNCS and Their Combinations

**Figure 2:**
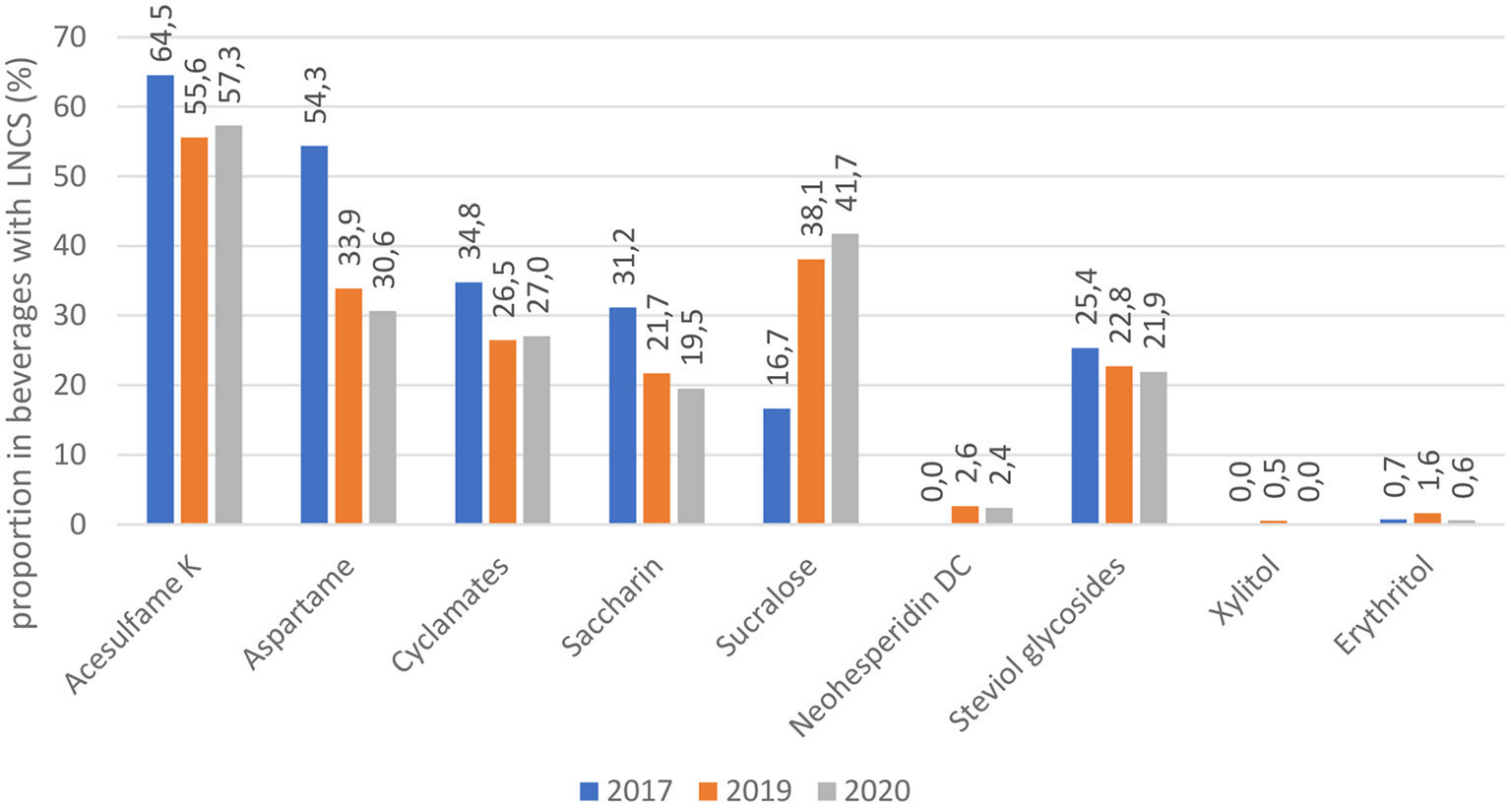
Frequency of different low and no-calorie sweeteners (LNCS) in beverages with LNCS [data for 2017 and 2019 from Hafner et. al (26)].

An overview of the use of specific sweeteners showed no significant differences in comparison with the previous data collection in 2019 (Figure 2). However, some interesting trends identified in a previous study (26) continued in 2020. We observed a further increase in the use of sucralose, and a consequently lower use of most other sweeteners. Consistent with the results from 2019, the most common sweetener in 2020 remained acesulfame K (*N* = 191; 57.3%), followed by sucralose (*N* = 139; 41.7%) and aspartame (*N* = 102; 30.6%).

In beverages with LNCS, the use of multiple sweeteners at the same time is still prevalent (65%), while the use of only one LNCS (35%) is limited to the use of steviol glycosides (*N* = 59), sucralose (*N* = 51), and saccharin (*N* = 6) (Supplementary Table 1). Even though steviol glycosides and saccharin were used as single LNCS, they were always combined with added sugar. Meanwhile, sucralose was used as single LNCS without added sugar in 16 products. Drinks with LNCS most commonly contained a mix of two LNCS (*N* = 128, 38.4%). The most common combination was acesulfame K and sucralose (*N* = 54), followed by the combination of acesulfame K and aspartame (*N* = 42). Interestingly when mixing three or more LNCS, acesulfame K was always present in the mixture. Even for products containing only two LNCS, products that did not contain acesulfame K were in the minority (*N* = 26), and were usually combined with added sugar or fruit juices/concentrates. The results also suggested that as the number of sweeteners increases, the frequency of added sugar decreases.

## Discussion

This study showed that the market for non-alcoholic beverages has changed rapidly in recent years. While a previous study found the first signs of growing use of LNCS in non-alcoholic beverages in Slovenia (26), this trend was clear when the latest data were taken into account. Our results showed a significant increase in the availability of beverages with LNCS, from 13.2% in 2017 and 15.5% in 2019, to 20.2% in 2020. In the period of 2017–2019 the changes mostly affected energy drinks; use of LNCS increased significantly. This trend stopped in 2020. On the other hand, in 2020 there was a marked growth in soft drinks with LNCS (from 19.6% in 2019 to 26.7% in 2020; *p* < 0.01), which is the most widespread category of non-alcoholic beverages in the Slovenian market.

We should note that the frequency of the use of LNCS in non-alcoholic beverages in Slovenia (20.2%) is still lower than in most other countries where it has been investigated (16, 37, 38). However, given the observed rapid changes, Slovenia is quickly approaching the proportion of beverages with LNCS in the US (23%; 2015–2017) (38) and Hong Kong (25%; 2019) (39). Even more frequent use of LNCS in beverages was reported in Spain (39%; 2013) (37), but the latter study was conducted with a very different methodological approach, using food consumption survey data. Very recently, a study with a methodological approach similar to our study was also conducted in Spain (15), but because only foods with LNCS were investigated, the proportion of use of LNCS was not calculated.

In our study, growth in the use of LNCS occurred particularly in the availability of beverages with both added sugar and LNCS. We also observed that increased sales-volume market-share for beverages with LNCS reflected the increase in the availability of such products. Increased sales for beverages with LNCS were also reported in a US study, which highlighted, that from 2002 to 2018, sales of products containing both added sugar and LNCS increased by almost 30% (40). The current study, as well as others (26, 38), observed that partially replacing sugar with LNCS can help reduce energy by almost half, while using only LNCS for sweetening leads to an even greater reduction. The increased supply of beverages with LNCS, therefore, impacted the overall mean EV and TSC, which decreased significantly from 2017 (*p* < 0.001). Encouragingly, the reduction of the average sugar content in the available non-alcoholic beverages was also influenced by the reduction of sugars in beverages sweetened only with added sugar (*p* < 0.01). This particularly applied to sugar-sweetened soft drinks.

Results of analyses of product-specific reformulations showed that 144 (16.8%) out of 859 matched products reduced their sugar content. Interestingly, a minority of these beverages were reformulated with the help of LNCS (*N* = 31; 3.6%). This is a promising result, since the Slovenian nutrition policy programme (28) encourages reformulation without LNCS, to reduce the preference for sweetness in the population. At the same time, it should be pointed out that products that used LNCS reduced their sugar content much more (−3.3 g per 100 ml) compared to those that did not use them (−1.1 g per 100 ml). However, the reformulated products still had a fairly high average sugar content (7.4 g per 100 ml), which was similar to the overall average (7.3 g), indicating that reformulation is particularly focused on products with a higher sugar content. Most of the reformulated beverages were soft drinks (*N* = 107), more precisely fruit drinks, which is the group of beverages that has been the most reformulated in Portugal in the past decade (41). It is also important to note that we also observed cases of increased TSC (*N* = 35), and those where added LNCS was not accompanied by lower TSC (*N* = 4). In such cases, the sweetness was increased without any health benefits, which is in clear conflict with public health goals.

Among the specific LNCS, the use of acesulfame K predominated in 2020, followed by sucralose and aspartame, which is aligned with the 2019 results. Most beverages contained multiple LNCS, while sole LNCS occurred mostly in beverages with sucralose and steviol glycosides, and occasionally saccharin. During recent years, the use of LNCS has shifted toward the increased use of sucralose and the decreased use of aspartame. Reduced occurrence of aspartame has also been observed in Portugal (41) and the US (40). Even though aspartame has been re-evaluated for safety (42), its use still remains controversial (43). This raises doubts in consumers, which is why manufacturers have replaced aspartame with novel, less notorious LNCS, such as sucralose and steviol glycosides (40). The use of sucralose is also increasing in Slovenia. In the US, the intake of sucralose increased by over 30% in a 16 year period, which could also be due to the increased supply of prepackaged products containing sucralose (40). Sucralose is relatively new to the market. Its use has rapidly increased because of its sucrose-like taste and good stability (44). Due to these properties, it appears both alone and in combination with other sweeteners. Increased use of sucralose has aroused the interest of researchers, who have begun to focus on its impact on health. Even though sucralose is currently considered safe, some studies indicate possible adverse effects on glucose metabolism, even in low amounts (15% of Acceptable Daily Intake, ADI) (45), and increased cardiovascular risk (46). A combination of carbohydrates and sucralose in beverages was highlighted as particularly risky (47). Interestingly, our study showed that use of steviol glycosides is slowly stagnating; they are only in fifth place among the most common sweeteners, used in 21.9% of beverages with LNCS. On the contrary, a considerable increase in the use of this sweetener has been reported from other countries (40, 41). In Chile, steviol glycosides are the second most used LNCS in the food supply, which has raised concerns that ADI value could be exceeded in some vulnerable groups, such as children (16), but a subsequent study indicated that this is not the case (48). A Portuguese study highlighted steviol glycosides as most commonly present in iced teas (41), which are a popular choice for children (49). Therefore, careful monitoring of this is crucial. In Slovenia, the frequency of use of steviol glycosides is currently relatively low, but given the rapid changes in LNCS use, this could change in the coming years. Steviol glycosides are commonly perceived by consumers as a natural sweetener, and are therefore rarely mixed with other LNCS (16, 50). However, the use of mixes of LNCS is still predominant in beverages. For the first time, we also reviewed LNCS combinations. We found out that combination of two LNCS is the most common, with acesulfame K and sucralose emerging as the most frequent blend. The same result was also reported in Spain, across the whole food supply (15). Blends can intensify the sweet taste of individual LNCS and prevent an unpleasant aftertaste (50). Our results indicated that with the increased number of LNCS, the frequency of added sugar has decreased. Therefore, beverages with only one LNCS in most cases also contained added sugars, while beverages with a blend of five LNCS did not contain any. This suggests that blends of LNCS could help to notably reduce sugar content in beverages.

It should be noted that in Slovenia beverages account for the largest share of sugar sold in the country (27), and are also the biggest contributor to free sugar intake (32% in adolescents, 34% in adults, and 31% in the elderly) (8). However, a reduction in TSC in beverages has occurred much slower than in some other countries, where sugar taxes have been implemented. For example, in Portugal, 1 year after tax implementation in 2017, 50% of soft drinks above the taxation level (8 g sugar/100 ml) reduced their TSC below this limit (51), and now only 15% exceed it (41). Meanwhile, in Slovenia (2020), about one third (*N* = 310; 34.5%) of soft drinks have a TSC above 8 g/100 ml. Although similar benefits of taxation have been seen in other countries, such reformulations might considerably increase the use of LNCS (52), and this could affect health risk analyses. At the same time, LNCS maintain or even intensify the sweetness of drinks, which hinders the main public health message to reduce the preferences for sweetness in our diets. Policy approaches for lowering the TSC in beverages are desirable, however, attention should be paid to possible excessive substitution of sugar with LNCS.

### Strengths and Limitations

As the main strength of this study, we should highlight the representativeness of the sample, which included beverages from all major retailers, representing a vast majority of the Slovenian food market. A repeated cross-sectional approach with the use of the same methodology and three time points allowed us to make meaningful comparisons and identify changes in the food supply. It is also noteworthy that there are only a few countries in Europe where the infrastructure enables such studies, which is why this study provided important insights to better understand the common European market. Some study limitations should be also mentioned. First, the data collection in 2020 included one discount retailer which was not included in monitoring in 2017/2019. We carefully checked that this did not have a major effect on the study results. In the whole 2020 dataset LNCS were present in 20.2% beverages; after exclusion of products found only at the additional retailer (*N* = 103), it was 19.8%. Another limitation is that sales data were not available for all beverages, however we covered 68% and 61% of the 2017 and 2020 sample, respectively. To exclude the possibility of the error due to the missing sales data, the offer of products included in sale-weighting were compared with the whole sample; no notable differences were observed between both samples. A limitation of the present study is also that all the information was extracted from food labels, which may differ from the actual chemical compositions of the beverages. We also only investigated the use of (declared) LNCS, and not their quantity, as this information is not indicated on the label. Finally, we used EAN barcodes for food matching in the analyses of product-specific reformulations. In case that the product has changed its EAN barcode, we were unable to match it with the previous formulation.

## Conclusions

This study explored the use of LNCS in non-alcoholic beverages in the 2020 edition of Slovenian branded foods database, for comparison with previous data. We showed that the use of LNCS in beverages increased for more than half (+53%)–from 13.2% in year 2017 to 20.2% in year 2020, with even more notable growth in soft drinks (for 59%–from 16.8% in 2017 to 26.7% in year 2020). Increased availability of beverages with LNCS also reflected in even higher increase in sales volumes; market-share of beverages with LNCS increased for 69%–from 10.8% in 2017 to 18.2% in year 2020. While study results also indicated some changes in the content of energy and sugars in both reformulated and newly launched beverages, most beverages in the Slovenian food supply still have very high energy/sugar content. To achieve public-health goals, more efficient reformulation activities are needed. Further monitoring of the composition of beverages in the food supply is needed also to assess the efficiency of such activities.

## Data Availability Statement

The raw data supporting the conclusions of this article will be made available by the authors, without undue reservation.

## Author Contributions

IP: conceptualization, and manuscript writing—review and editing. EH: data analyses, formal analysis, writing—original draft preparation. IP and EH: methodology. All authors have read and agreed to the published version of the manuscript.

## Funding

Data collection for this study was supported by the national research programme Nutrition and Public Health (P3-0395, funded by the Slovenian Research Agency) and research project L3-9290, funded by the Ministry of Health of Republic of Slovenia and Slovenian Research Agency. The study was conducted within the Food Nutrition Security Cloud project (FNS-Cloud), which received funding from the European Union's Horizon 2020 Research and Innovation programme (H2020-EU.3.2.2.3.—A sustainable and competitive agri-food industry) under grant agreement no. 863059. Information and views in this report do not necessarily reflect the official opinion or position of the European Union. Neither European Union institutions and bodies, nor any person acting on their behalf, may be held responsible for the use that may be made of the information contained herein.

## Conflict of Interest

The authors declare that the research was conducted in the absence of any commercial or financial relationships that could be construed as a potential conflict of interest.

## Publisher's Note

All claims expressed in this article are solely those of the authors and do not necessarily represent those of their affiliated organizations, or those of the publisher, the editors and the reviewers. Any product that may be evaluated in this article, or claim that may be made by its manufacturer, is not guaranteed or endorsed by the publisher.
