# Supplementary material for: The Sharp Rise in the Use of Low- and No-Calorie Sweeteners in Non-Alcoholic Beverages in Slovenia: An Update Based on 2020 Data
Source: Front Nutr. 2021 Nov 19;8:778178. doi: 10.3389/fnut.2021.778178 (PMC8640248; doi:10.3389/fnut.2021.778178)
Supplement: Supplementary file 1 [file Data_Sheet_1.docx]

Supplementary Material

Supplementary Figure S1: Comparison of the distribution of available and sale-weighted non-alcoholic beverages, based on the [a] added sugar and/or [b] low and no-calorie sweeteners (LNCS).

Notes: a─added sugar, b─added LNCS; sale-weighted distribution correspond to sales-volume market-shares

Supplementary Table 1: Number and types of low and no-calorie sweeteners (LNCS) present in non-alcoholic beverages with added LNCS (N=333) in Slovenian food supply (2020), and their combination with added sugar.

| **Number of**  **different**  **LNCS** | **Type of LNCS** | **All**  **(N, %)** | **No added sugar**  **(N, %)** | **Added sugar**  **(N, %)** |
| --- | --- | --- | --- | --- |
| **1** |  | **116 (34.8%)** | **16 (4.8%)** | **100 (30%)** |
|  | Saccharin | 6 (1.8%) | 0 (0%) | 6 (1.8%) |
|  | Steviol glycosides | 59 (17.7%) | 0 (0%) | 59 (17.7%) |
|  | Sucralose | 51 (15.3%) | 16 (4.8%) | 35 (10.5%) |
| **2** |  | **128 (38.4%)** | **66 (19.8%)** | **62 (18.6%)** |
|  | Acesulfame K + Aspartame | 42 (12.6%) | 22 (6.6%) | 20 (6%) |
|  | Acesulfame K + Saccharin | 6 (1.8%) | 0 (0%) | 6 (1.8%) |
|  | Acesulfame K + Sucralose | 54 (16.2%) | 37 (11.1%) | 17 (5.1%) |
|  | Cyclamates + Saccharin | 12 (3.6%) | 0 (0%) | 12 (3.6%) |
|  | Cyclamates + Sucralose | 1 (0.3%) | 0 (0%) | 1 (0.3%) |
|  | Saccharin + Sucralose | 1 (0.3%) | 1 (0.3%) | 0 (0%) |
|  | Sucralose + Erythritol | 2 (0.6%) | 0 (0%) | 2 (0.6%) |
|  | Sucralose + Steviol glycosides | 10 (3%) | 6 (1.8%) | 4 (1.2%) |
| **3** |  | **60 (18%)** | **44 (13.2%)** | **16 (4.8%)** |
|  | Acesulfame K + Aspartame + Cyclamates | 24 (7.2%) | 23 (6.9%) | 1 (0.3%) |
|  | Acesulfame K + Aspartame + Neohesperidin DC | 5 (1.5%) | 0 (0%) | 5 (1.5%) |
|  | Acesulfame K + Aspartame + Saccharin | 1 (0.3%) | 1 (0.3%) | 0 (0%) |
|  | Acesulfame K + Aspartame + Sucralose | 4 (1.2%) | 4 (1.2%) | 0 (0%) |
|  | Acesulfame K + Cyclamates + Saccharin | 13 (3.9%) | 3 (0.9%) | 10 (3%) |
|  | Acesulfame K + Cyclamates + Sucralose | 11 (3.3%) | 11 (3.3%) | 0 (0%) |
|  | Acesulfame K + Sucralose + Steviol glycosides | 2 (0.6%) | 2 (0.6%) | 0 (0%) |
| **4** |  | **26 (7.8%)** | **21 (6.3%)** | **5 (1.5%)** |
|  | Acesulfame K + Aspartame + Cyclamates + Saccharin | 26 (7.8%) | 21 (6.3%) | 5 (1.5%) |
| **5** |  | **3 (0.9%)** | **3 (0.9%)** | **0 (0%)** |
|  | Acesulfame K + Cyclamates + Sucralose + Neohesperidin DC + Steviol glycosides | 3 (0.9%) | 3 (0.9%) | 0 (0%) |
